# Hydroxycinnamic acid derivatives for UV-selective and visibly transparent dye-sensitized solar cells

**DOI:** 10.1038/s41598-022-17236-6

**Published:** 2023-02-24

**Authors:** Arum Dista Wulansari, Dini Hayati, Dang Xuan Long, Kyungah Choi, Jongin Hong

**Affiliations:** 1 Department of Chemistry, Chung-Ang University, 84 Heukseok-ro, Dongjak-gu, Seoul, 06975 Republic of Korea; 2 Department of Smart Cities, Chung-Ang University, 84 Heukseok-ro, Dongjak-gu, Seoul, 06974 Republic of Korea; 3 Department of Interior Architecture Design, Hanyang University, 222 Wangsimni-ro, Seongdong-gu, Seoul, 04763 Republic of Korea

**Keywords:** Chemistry, Energy, Solar cells

## Abstract

Naturally abundant dyes are very attractive for the development of dye-sensitized solar cells (DSSCs). Hydroxycinnamic acid derivatives, such as caffeic acid (**CA**), ferulic acid (**FA**), and *p*-coumaric acid (**PA**), were considered for the selective harvesting of ultraviolet A (UVA) (315–400 nm) photons. Their spectroscopic and electrochemical properties were investigated both theoretically and experimentally. They were further successfully adopted as photosensitizers in UV-selective and visibly transparent DSSCs, which exhibited a power conversion efficiency of 0.22–0.38% under AM (air mass) 1.5G (global) illumination (100 mW/cm^2^) and 3.40–3.62% under UVA irradiation (365 nm, 115.22 mW/cm^2^), with a corresponding visible light transmittance (VLT) of 49.07–43.72% and a general color rendering index (*R*_*a*_) of 93–90.

## Introduction

As the global urban population grows rapidly, energy consumption in cities has become a major cause of climate change. The integration of renewable energy in cities can provide a transition to greener city environments. Importantly, buildings and construction accounted for 36% of global energy demand and 37% of energy-related carbon dioxide (CO_2_) emissions in 2020^1^. To mitigate this energy consumption, the concept of zero-energy building (ZEB) has been introduced, where the energy generated on-site is equal to or more than the energy delivered from supply grids. Implementation of ZEB has become obligatory for new residential and commercial construction^2^, and a promising way of achieving this is Building-Integrated Photovoltaics (BIPV), in which photovoltaic modules are mounted onto the building envelopes.

Over the years, advancements in glazing technologies associated with modern building architecture have led to the development of glass cladding systems, such as curtain walls, window walls, and key building envelope systems. Although the extensive use of glass cladding systems is responsible for cooling loads in summer and heating loads in winter, photovoltaic (PV) curtains and window walls are crucial for next-generation glazing technology. Unfortunately, the current challenge associated with PV glazing is an inherent trade-off between transmittance and power conversion efficiency (PCE) because conventional solar cells absorb visible light to produce electricity. Recently, wavelength-selective technologies have emerged; these technologies use excitonic materials that selectively absorb ultraviolet (UV)^3–6^ or near-infrared (NIR) light^7–11^ and such wavelength-selective PVs are anticipated to overcome the limitations of semi-transparent and colored PVs for BIPV applications.

Among emergent photovoltaic technologies, dye-sensitized solar cells (DSSCs) are especially favorable to achieve high transparency in the visible light region because of the wavelength-selective absorption of photosensitizers and the use of optically transparent conductive oxide substrates^12–16^. The synthetic photosensitizers are expensive and environmentally unfriendly, and thus naturally abundant dyes should be considered to replace expensive chemical synthesis processes with simple extraction processes. To date, naturally available dyes, such as anthocyanin, betalains, flavonoids, carotenoid, and chlorophyll, have been used to fabricate DSSCs^17–19^ and representative results can be found in References 18 and 19. Most of natural dyes show absorption from 400 to 700 nm in the visible light regime. For example, anthocyanins exhibit high absorption at long wavelengths (e.g. 580–700 nm). Betacyanins and betaxanthins absorb in the range from 400 to 600 nm. Chlorophyll absorbs all wavelengths of visible light except green. Unfortunately, natural dyes, which selectively harvest UV photons, have not been much investigated in the dye-sensitized applications. In this study, we introduce hydroxycinnamic acids (HCAs) to the wavelength-selective technology as potential UV-absorbing photosensitizers. HCAs possess a chemical backbone comprising nine carbon atoms (C6–C3) and are the major subgroup of phenolic acids with ubiquitous distribution in the plant kingdom^20^. HCAs, such as caffeic acid (**CA**), ferulic acid (**FA**) and *p*-coumaric acid (**PA**), are natural phenolic compounds that are abundantly found in tea leaves, coffee, fruits, vegetables, and whole grains^21,22^. Interestingly, they have a specific structure in which a carboxyl group is separated from an aromatic ring by a double bond, forming a π-electron system. Theoretically and experimentally, they are known to exhibit strong absorbance in the UV region (220–400 nm)^20,23–25^. Therefore, we investigated the efficacy of HCAs for harvesting UV photons and demonstrated UV-selective and visibly transparent DSSCs.

## Results and discussion

### Theoretical calculations and characterization

**Figure 1:**
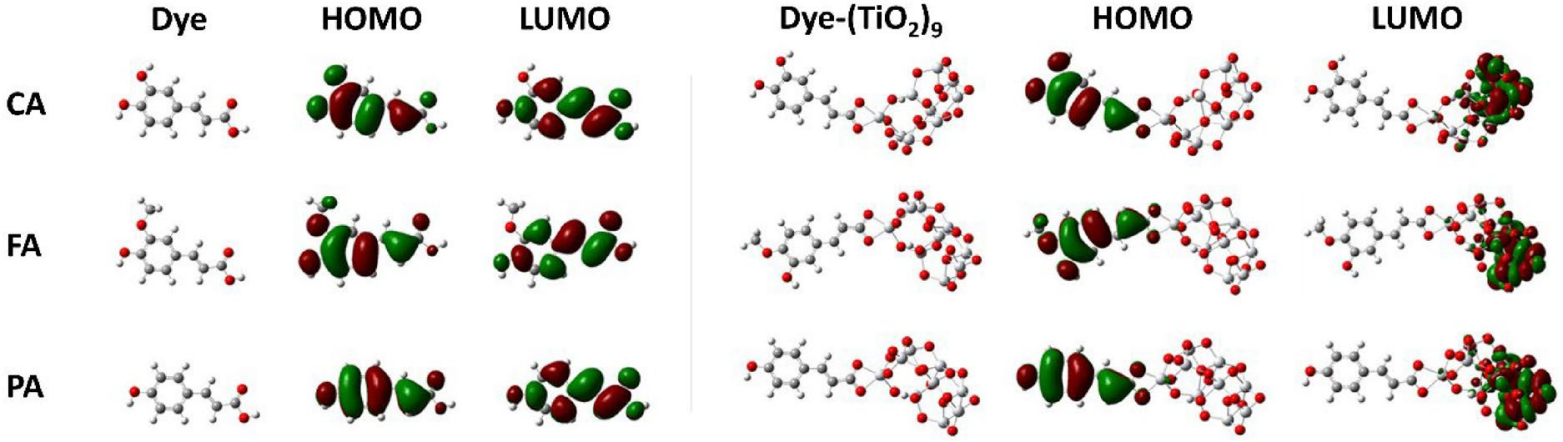
Optimized structures and frontier molecular orbitals (FMOs) of isolated dyes (CA, FA, PA) and dye-(TiO_2_)_9_ complexes.

Density functional theory (DFT) and time-dependent density functional theory (TDDFT) calculations were performed to gain insight the structural, electronic structures and optical property of **CA**, **FA**, and **PA** before and after binding to TiO_2_ cluster. Figure 1 shows the optimized geometries and frontier molecular orbitals (FMOs) of the HCAs and HCA-TiO_2_ complexes, respectively. In the optimized structures, the double bond (–C=C–) links the aromatic ring with the carboxylic acid (–COOH), and thus, all compounds present a completely planar structure. The FMOs provide valuable information for predicting the optical and electronic properties of molecules. All the HCAs and HCA-TiO_2_ complexes exhibited similar FMO spatial distributions. The presence of two opposing electric dipoles, viz. –C=C– and carboxyl groups, disturbed the π-electron system in the HCAs, and the electrons of the free HCAs were spread across the entire molecule with the largest contribution from the aromatic ring at the highest occupied molecular orbital (HOMO) and the lowest unoccupied molecular orbital (LUMO) levels. Unlike the free HCAs, the electrons of the HCA-TiO_2_ complexes were entirely concentrated on TiO_2_ at the LUMO level. This indicates that the excited electrons could be easily injected into TiO_2_ via the carboxylic unit.

**Figure 2:**
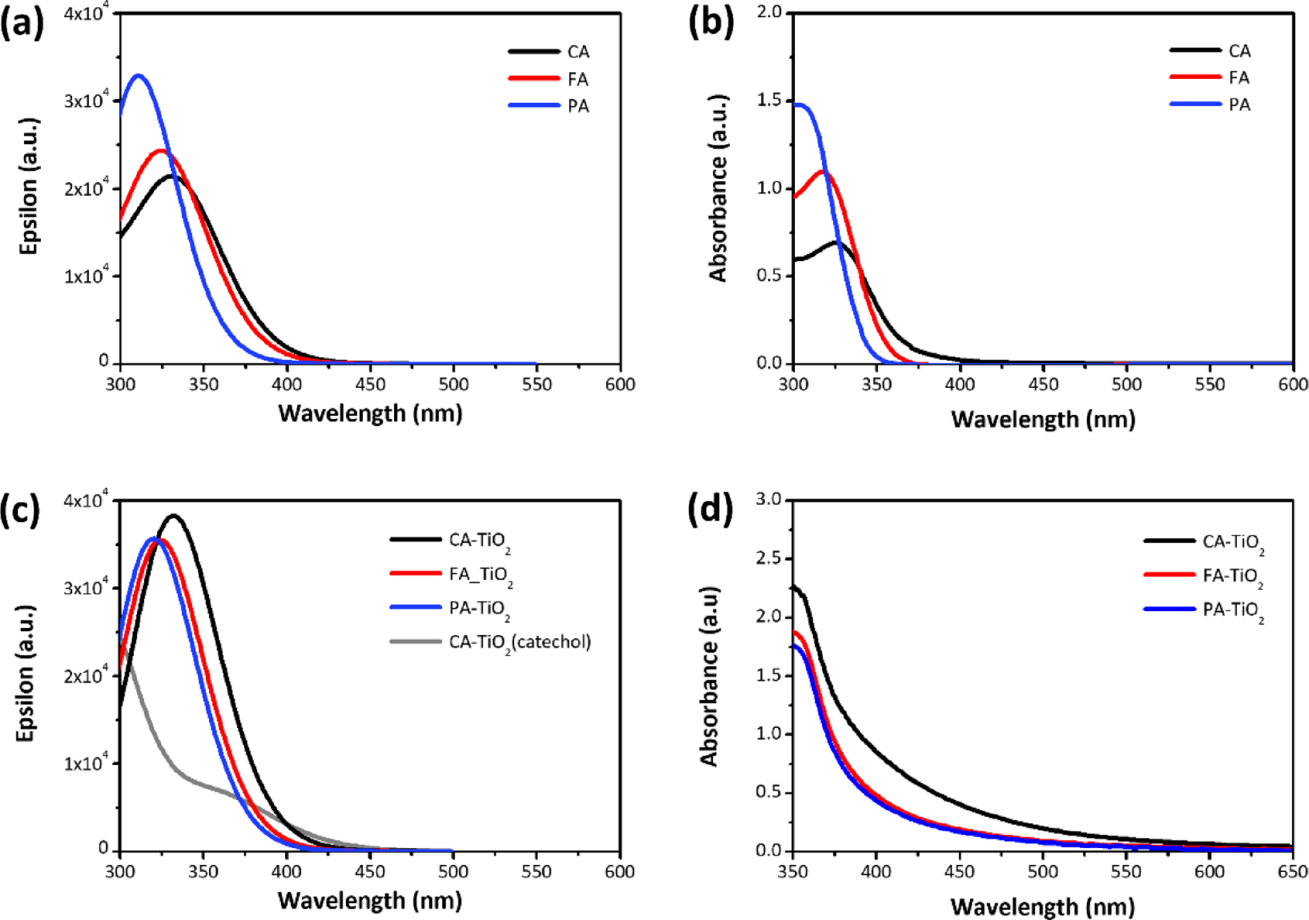
Calculated UV–visible spectra of (**a**) HCAs and (**c**) HCA-TiO_2_ complexes and experimental UV–visible spectra of (**b**) HCAs and (**d**) HCA-grafted mesoporous TiO_2_ films.

Figure 2a,b show the simulated and experimental UV–visible spectra of the HCAs, respectively. Prominent absorption peaks of pure HCAs, dissolved in EtOH at a concentration of 5 × 10^–4^ M, were observed in the UVA region (315–400 nm). The maximum absorbance peak is due to the dominant transition from HOMO to LUMO, which can be ascribed to the π–π* transition of the aromatic moiety^26^. The molar extinction coefficients of **PA**, **FA**, and **CA** were 2969.06, 2189.18, and 1381.74 M^−1^ cm^−1^, respectively, and the corresponding optically determined bandgaps were 4.26, 4.07, and 4.09 eV. Interestingly, the spectrum of **PA** differed from those of **FA** and **CA** because the main absorption band corresponds to the aromatic ring with less substitutions^20^, which results in hypsochromic shift and high peak intensity. These differences can be rationalized by the *para* substitution of the aromatic rings in **FA** and **CA**, which would decrease the conjugation between the carboxyl group and the ring^27^. Figure 2c,d show simulated and experimental UV–visible spectra of HCA-TiO_2_ complexes, respectively. The broadening and red-shift of the peaks after binding to TiO_2_ is due to the electronic coupling between the carboxyl group and TiO_2_, and this results in reduced LUMO energies. Interestingly, CA exhibited a broader and stronger absorption in the visible light regime than PA and FA. This can be due to a direct charge-transfer excitation from the catechol π level to the bottom of the TiO_2_ conduction band^28^ because CA has both catechol and carboxylic acid groups that are common dye anchors in DSSCs^29^. In addition, the strength of the interaction between the dye molecule and the TiO_2_ cluster (i.e., adsorption energy) was calculated and summarized in Table S1. A large negative value indicates more efficient charge transfer from the dye to the TiO_2_ conduction band.

**Figure 3:**
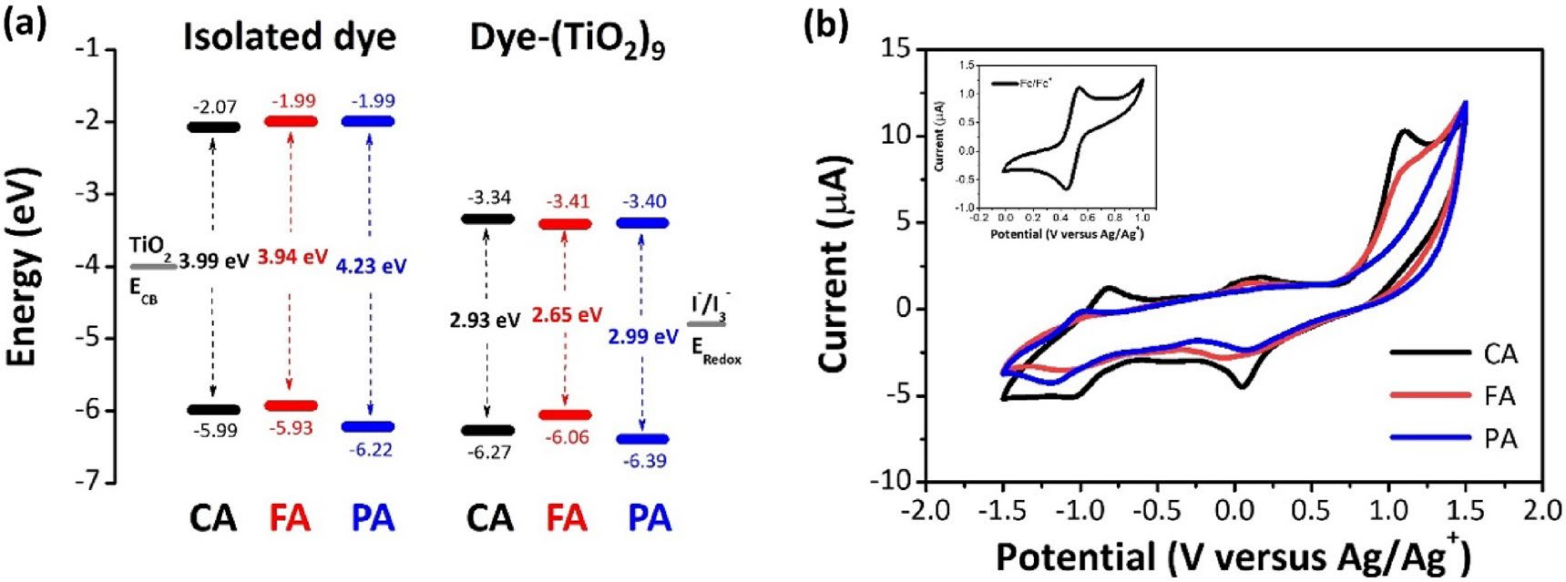
(**a**) Calculated energy levels of the HCA derivatives (PA, FA, CA) and HCA-grafted TiO_2_ complexes and (**b**) cyclic voltammograms of the synthesized dyes in EtOH (1 mM) at a scan rate of 100 mV s^−1^. Ferrocene/ferrocenium (Fc/Fc^+^) couple was used as an external reference (Inset).

Figure 3a shows the calculated molecular orbital energies of the HOMO and LUMO levels of the HCAs and HCA-TiO_2_ complexes. The structural difference had a substantial influence on HOMO levels rather than the LUMO. The bidentate chelation resulted in the stabilization of the LUMO and thus a decrease in the HOMO–LUMO energy gap. The calculated HOMO energies were lower than the redox potential of ${I}^{-}/{I}_{3}^{-}$ (− 4.80 eV)^30^, which ensures that the oxidized dyes can regain the electrons from the iodide ions and be regenerated. The calculated LUMO energies were higher than that of the conduction band (CB) of TiO_2_ (− 4.00 eV)^30^, indicating that the excited-state dyes can easily inject electrons into the TiO_2_ CB. Cyclic voltammetry (CV) was used to determine the electrochemical characteristics and HOMO–LUMO energy levels of all the compounds, and their CV plots are shown in Fig. 3b. HOMO levels were calculated from the first oxidation potentials (*E*_*ox*_) of the dyes, while LUMO levels were obtained from the difference between the *E*_*ox*_ and excitation transition energies (*E*_*0−0*_). Table 1 lists the photophysical and electrochemical properties of the HCAs. It showed that the aromatic ring with more substitutions in **CA** and **FA** led to a reduction of the HOMO–LUMO gap energy due to stabilization of the HOMO and LUMO energy levels.

**Table 1:** Photophysical and electrochemical properties of CA, FA, and PA.

Dye	*λ*_*max*_^a^ (nm)	ε^b^ (M^−1^ cm^−1^)	*λ*_*edge*_^c^ (nm)	*E*_*gap*_^d^ (eV)	*E*_*ox*_^e^ (V)	HOMO^f^ (eV)	LUMO^g^ (eV)
CA	325	1381.74	379	3.27	0.75	− 5.15	− 1.88
FA	316	2189.18	360	3.44	0.71	− 5.11	− 1.67
PA	298	2969.06	345	4.59	0.68	− 5.08	− 0.49

^a^Absorption maximum in EtOH solutions (10^–5^ M).

^b^Molar extinction coefficient, calculated using Lambert–Beer’s law.

^c^The onset of absorption spectra in EtOH solutions (10^–5^ M).

^d^*E*_*gap*_ was estimated from the onset of the absorption spectra in EtOH, *E*_*gap*_ = 1240/*λ*_*edge*_*.*

^e^*E*_*ox*_ was measured by CV in EtOH and ferrocene was used as an external reference.

^f^Calculated from HOMO = − (E_ox_ + 4.4)eV.

^g^Calculated from LUMO = *E*_*gap*_ + HOMO.

### Photovoltaic performance and transparency

**Figure 4:**
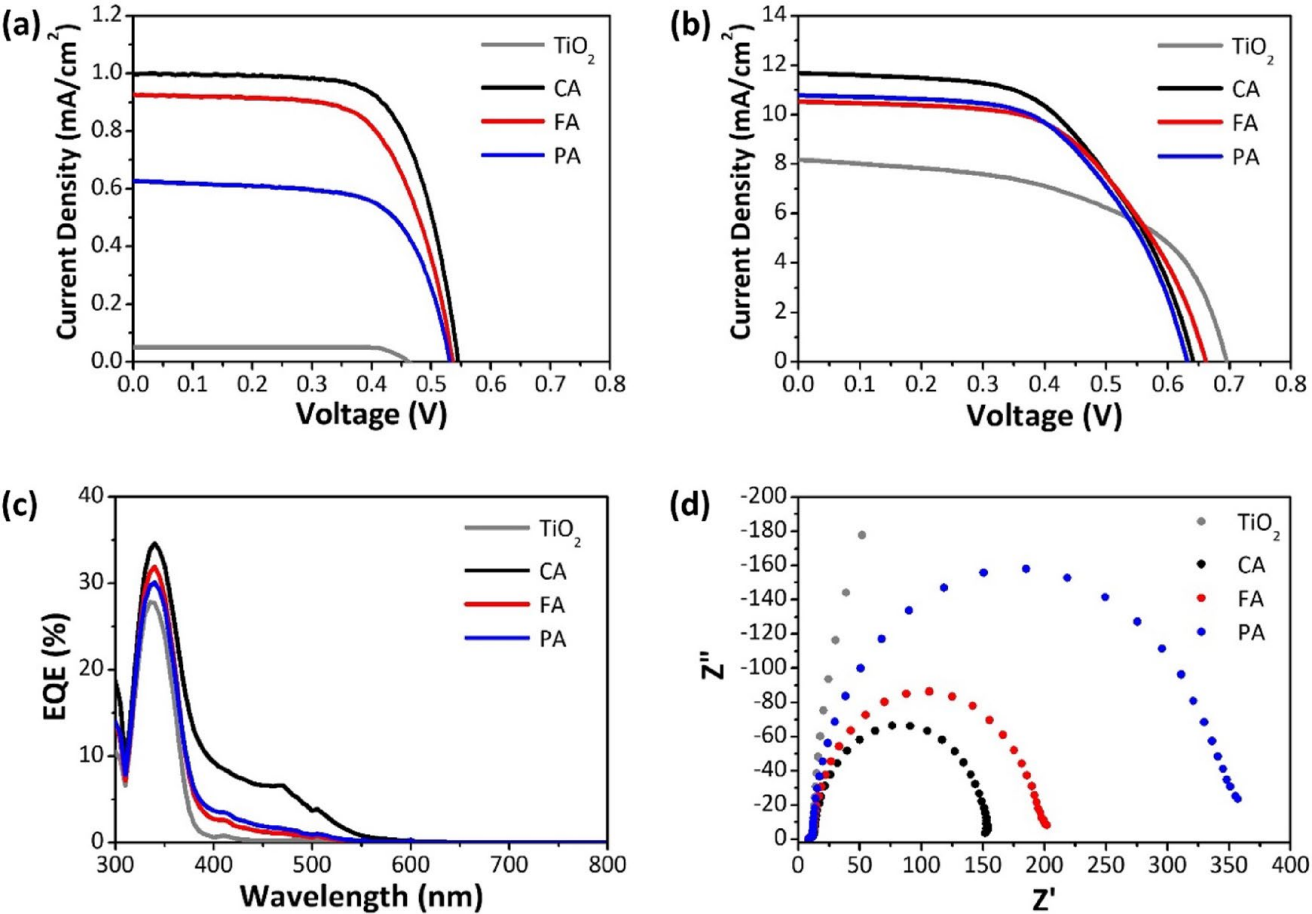
J-V characteristics under (**a**) one-sun illumination (AM 1.5G, 100 mW/cm^2^) and (**b**) UVA radiation (365 nm, 115 mW/cm^2^), (**c**) EQE of DSSCs sensitized with CA, FA, and PA, (**d**) Nyquist plots of DSSCs under one-sun illumination.

The photovoltaic performance was investigated under two different light sources, viz. AM 1.5G simulated light (100 mW/cm^2^) and UV LEDs (365 nm, 115 mW/cm^2^), as shown in Fig. 4a,b, respectively. The photovoltaic parameters are listed in Table 2. UV radiation is approximately 5% of solar terrestrial radiation, and thus DSSCs sensitized with HCAs exhibit low PCEs under one-sun illumination. However, HCAs can harvest UV photons and thus allow the production of electricity under monochromatic UVA irradiation. It should be noted that the cell with only TiO_2_ and an iodide/triiodide redox couple could produce electricity under UVA illumination. This indicates that TiO_2_ absorbed photons from UVA irradiation and generated free electrons for the DSSC operation. Table S2 summarizes the photovoltaic parameters as a function of the UVA intensity. As the intensity increased, open-circuit voltage (*V*_*oc*_) leveled off and short-circuit current density (*J*_*sc*_) steadily increased, but the PCE reached a peak. Among the parameters, *J*_*sc*_ is strongly correlated with the light harvesting efficiency (LHE), which depends on the extinction coefficient of the molecule. It is obtained by integrating the product of the incident photon flux density and the external quantum efficiency (EQE) of the cell throughout the wavelength range. The performance of the DSSCs were also measured after a period of aging at room temperature and in an open circuit (Fig. S1), and the photovoltaic parameters at 1 sun and UVA are summarized in Tables S3 and S4, respectively. Over 10 days, the **CA** (TiO_2_ only) cells sealed with thermoplastic Surlyn® sealants, which had some permeability towards oxygen and moisture^31^, lost 24% (55%) at 1 sun and 16% (40%) at UVA, respectively. Figure 4c shows the EQE spectra of the DSSCs sensitized with HCAs. The significant increase in *J*_*sc*_ of the **CA** cell relative to the **FA** and **PA** cells can be ascribed to its ability to collect more photons in the UVA and visible regions. However, the **FA** and **PA** cells selectively absorbed the UVA light and thus could be more visibly transparent than the **CA** cell. Additionally, EIS analysis was carried out to investigate the interfacial charge transfer processes in the DSSC. Nyquist plots of the DSSCs under standard illumination condition (AM 1.5G, 100 mW/cm^2^) are shown in Fig. 4d. The two semicircles indicating the charge transfer behaviors in the intermediate- and high-frequency regions can be attributed to the electron transfer at the TiO_2_/dye/electrolyte interface and electrochemical charge transfer at the Pt/electrolyte interface, respectively. The large semicircles in the intermediate-frequency region imply the large charge transfer resistance (*R*_*tr*_) under one-sun illumination. This agrees with the photovoltaic performance observed above. The EIS measurements were also repeated to examine the electrochemical stability for the as-fabricated and aged DSSCs at room temperature (Fig. S2). The semicircle in the intermediate-frequency region became larger over 10 days and thus the performance degradation was related to the TiO_2_/dye/electrolyte interface.

**Table 2:** Photovoltaic performance of DSSCs sensitized with CA, FA, and PA under one-sun illumination (AM 1.5G, 100 mW/cm^2^) and UVA radiation (365 nm, 115 mW/cm^2^).

Cell	*V*_*oc*_ (V)	*J*_*sc*_ (mA/cm^2^)	*FF* (%)	PCE (%)
**One sun illumination (AM 1.5G, 100 mW/cm**^**2**^**)**				
TiO_2_ only	0.463 ± 0.003	0.05 ± 0.26	86.58 ± 0.63	0.02 ± 0.01
CA	0.545 ± 0.003	1.00 ± 0.02	69.24 ± 0.69	0.38 ± 0.01
FA	0.536 ± 0.002	0.93 ± 0.01	67.68 ± 0.37	0.34 ± 0.01
PA	0.532 ± 0.005	0.63 ± 0.01	67.46 ± 0.49	0.22 ± 0.01
**UVA radiation (365 nm, 115 mW/cm**^**2**^**)**				
TiO_2_ only	0.696 ± 0.004	8.18 ± 0.03	54.85 ± 0.69	2.71 ± 0.05
CA	0.642 ± 0.073	11.68 ± 0.95	55.62 ± 0.61	3.62 ± 0.03
FA	0.662 ± 0.011	10.54 ± 0.15	56.97 ± 0.53	3.45 ± 0.02
PA	0.631 ± 0.087	10.79 ± 0.12	57.48 ± 0.40	3.40 ± 0.08

**Figure 5:**
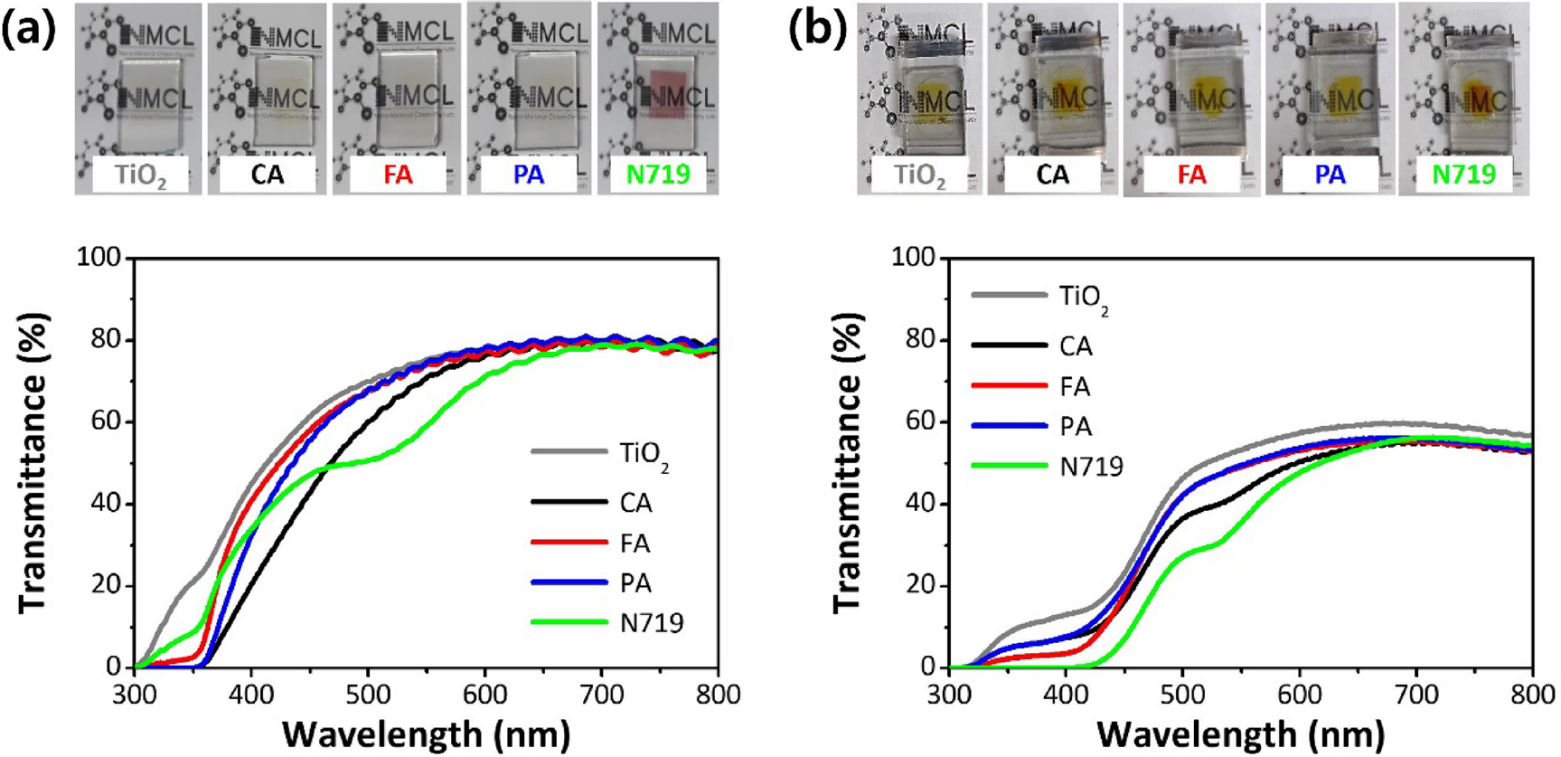
Photographs and transmittance spectra of (**a**) dye-grafted films and (**b**) DSSCs.

To realize visibly transparent and colorless solar cells, we should consider an inherent trade-off between PCE and visible light transmittance (VLT), where VLT approaches 100% as PCE approaches zero. Generally, the human eye is sensitive to light between 400 nm (violet) and 700 nm (red); thus, the device transparency (${\uptau }_{\mathrm{v}}$) should be evaluated by the ISO standard method (ISO 9050:2003) and the integration of the transmittance spectrum against human photopic vision, using the following Equation^32^:

$${\uptau }_{\mathrm{v}}=\frac{{\sum }_{380\mathrm{ nm}}^{780\mathrm{ nm}}\uptau \left(\uplambda \right){\mathrm{D}}_{\uplambda }(\uplambda )\mathrm{V}\left(\uplambda \right)\Delta\uplambda }{{\sum }_{380\mathrm{ nm}}^{780\mathrm{ nm}}{\mathrm{D}}_{\uplambda }\mathrm{V}\left(\uplambda \right)\Delta\uplambda }$$

where $\uptau \left(\uplambda \right)$, ${\mathrm{D}}_{\uplambda }$, $\mathrm{V}\left(\uplambda \right)$, and $\Delta\uplambda$ are the spectral transmittance of the device, the relative spectral distribution of illuminant D65, the CIE spectral luminosity function for photopic vision, and the wavelength interval, respectively. Figure 5a,b show the transmittance spectra of the HCA-grafted TiO_2_ films and the DSSCs sensitized with HCAs. The calculated VLT values are presented in Table 3. The films and cells exhibited low transmittance at wavelengths shorter than 400 nm. Their transmittance increased rapidly in the visible region and reached 80% of the films and 50% of the cells at wavelengths longer than 550 nm. These results confirm that HCAs can be used for UV-selective transparent photovoltaics (TPVs). Unfortunately, the decrease in transmittance after cell assembly is related to the transmission losses due to the Pt-based electrocatalyst and the iodide electrolyte^33^. In particular, the ${I}^{-}/{I}_{3}^{-}$ redox electrolyte absorbs many photons in the wavelength range of 300–500 nm.

**Table 3:** VLT (%) values of dye-grafted films and DSSCs.

	TiO_2_ only	CA	FA	PA	N719
Photoanodic film	74.89	70.04	73.41	74.03	61.29
Solar cell	52.85	43.72	48.63	49.07	37.47

### Color analysis

For color analysis, the spectrum of the light transmitted through the specimen (*S*(λ)) was calculated using the transmittance spectrum *T*(λ) and AM 1.5G (light source) spectrum *I*(λ), using the formula, *S*(λ) = *T*(λ)*I*(λ). According to the CIE recommendation^34,35^, the transmitted spectrum *S*(λ) was then used to compute the CIE tristimulus values as

$$\begin{aligned} & X = \int_{{380\, {\text{nm}}}}^{780\, nm} {S\left(\uplambda \right)\overline{x}\left(\uplambda \right){\text{d}}\uplambda } \\ & Y = \int_{380\, nm}^{{780\, {\text{nm}}}} {S\left(\uplambda \right)\overline{y}\left(\uplambda \right){\text{d}}\uplambda } \\ & Z = \int_{{380\, {\text{nm}}}}^{780\, nm} {S\left(\uplambda \right)\overline{z}\left(\uplambda \right){\text{d}}\uplambda } \\ \end{aligned}$$

where $\overline{x }\left(\uplambda \right)$, $\overline{y }\left(\uplambda \right)$, and $\overline{z }\left(\uplambda \right)$ are the color-matching functions of the CIE 1931 standard observer. The CIE 1931 (*x*, *y*) chromaticity coordinates are defined as:

$$x=X/(X+Y+Z) \mathrm{and} y=Y/(X+Y+Z)$$

Even though CIE 1931 chromaticity is most used for colorimetric specification, the color space is perceptually non-uniform^36,37^. The CIE 1976 uniform chromaticity scale was therefore constructed by mathematically converting the *x*, *y* chromaticity coordinates to *u′*, *v′* as:

$$u^{\prime} = 4x/\left( {12y - 2x + 3} \right)\, {\text{and}} \, v^{\prime} = 9y/\left( {12y - 2x + 3} \right)$$

**Figure 6:**
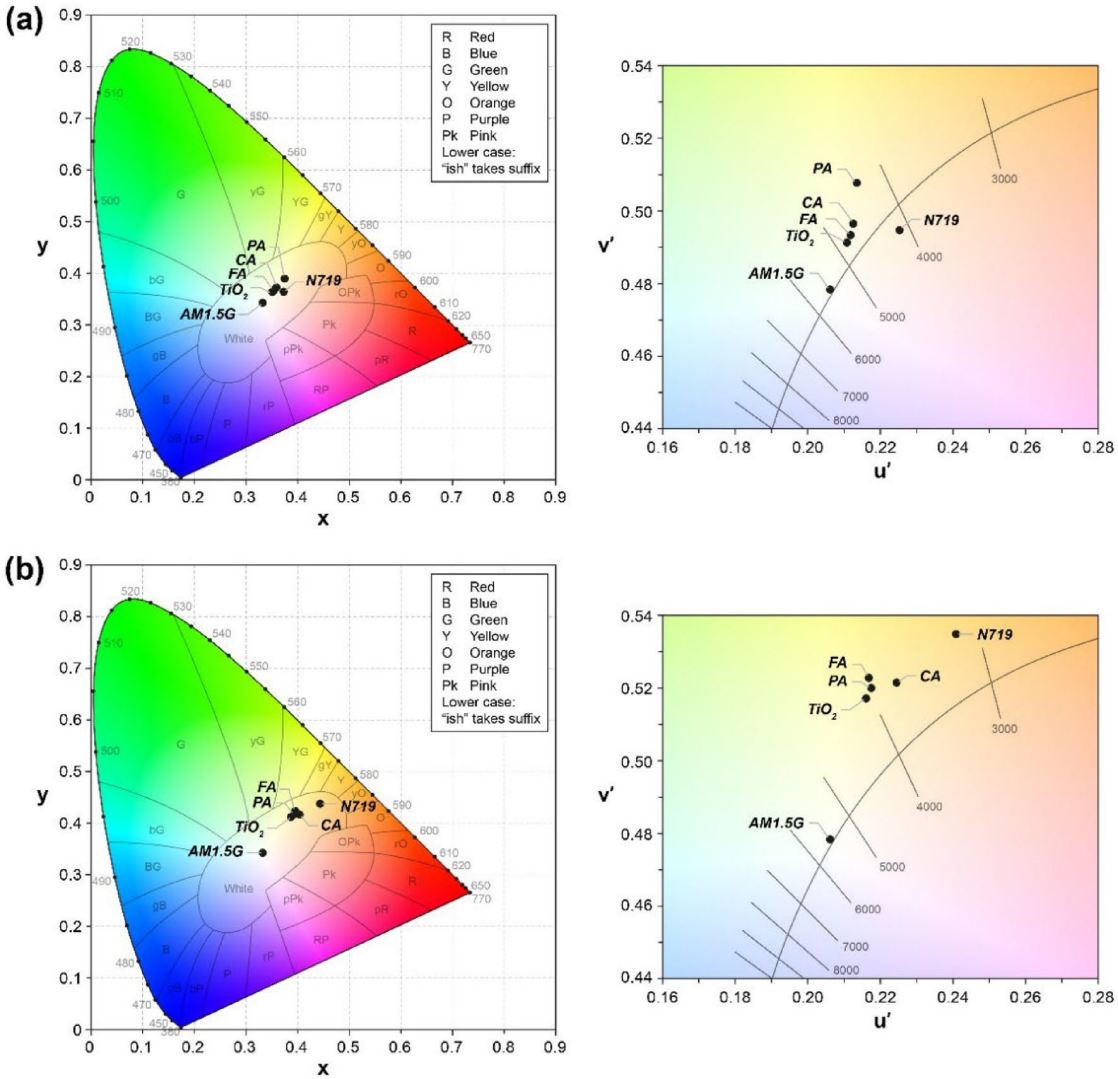
Color coordinates of (**a**) photoanodic films (TiO_2_-only, CA, FA, PA, and N719) and (**b**) their solar cells plotted on the CIE 1931 (*x*, *y*) and the CIE 1976 (*u*′, *v*′) chromaticity diagram. AM 1.5G is also included as the reference light source.

In Fig. 6, the color coordinates of the transmitted light are plotted on the CIE 1931 (*x*, *y*) and CIE 1976 (*u′*, *v′*) chromaticity diagram. The coordinates of the AM 1.5G light were also included as references. The CIE 1976 chromaticity is perceptually uniform and therefore, is more suited for estimating the magnitude of the color difference between cells.

The CIE colorimetry system was then used to calculate two figures of merit for describing the color qualities of transmitted light: correlated color temperature (CCT) and color difference. CCT is a measure of light color appearance, defined as the temperature of a blackbody radiator whose chromaticity point is closest to the chromaticity point of the light. A low CCT (less than 3500 K) corresponds to orangish light, whereas a high CCT (greater than 5500 K) corresponds to bluish light^38^. Next, the chromaticity difference from the original light source (AM 1.5G) was computed to characterize how the light changed as it was transmitted through the specimen. In the CIE 1976 (*u′*, *v′*) chromaticity, the Euclidian distance (Δ*u′v′*) between the chromaticity coordinates of the transmitted light and the AM 1.5G light (*u′* = 0.2062, *v′* = 0.4783) was calculated. Table 4 lists the chromaticity coordinates (*x*, *y*), (*u′*, *v′*), CCT, and color difference (Δ*u′v′*) of the transmitted light.

**Table 4:** Chromaticity coordinates (*x*, *y*), (*u*′, *v*′), CCT, and color difference (Δ*u*′*v*′) of the light transmitted through the TiO_2_-only, CA, FA, PA, and N719 photoanodic films and their solar cells.

Dye	*x*	*y*	*u*′	*v*′	CCT (K)	Δ*u'v'*
**Photoanodic films**						
TiO_2_	0.3511	0.3635	0.2109	0.4912	4825	0.0138
CA	0.3587	0.3723	0.2125	0.4964	4615	0.0192
FA	0.3545	0.3668	0.2119	0.4933	4724	0.0160
PA	0.3751	0.3894	0.2168	0.5062	4229	0.0299
N719	0.3730	0.3638	0.2254	0.4946	4116	0.0252
**Dye-sensitized solar cells**						
TiO_2_	0.3872	0.4116	0.2161	0.5170	4063	0.0400
CA	0.4038	0.4168	0.2245	0.5214	3729	0.0469
FA	0.3953	0.4234	0.2169	0.5227	3953	0.0457
PA	0.3927	0.4170	0.2176	0.5199	3970	0.0432
N719	0.4435	0.4375	0.2409	0.5348	3148	0.0663

In the case of the films, the CCTs of the **CA** and **FA** cells were 4615 K and 4724 K, respectively, which were relatively close to that of the **TiO**_**2**_**-only** cell (4825 K). In contrast, the CCTs of the **PA** and **N719** cells were low, at 4229 K and 4116 K, respectively. As for the color difference, the deviation from the original light source was smallest for the **FA** (0.0160) and increased in the order of **CA** (0.0192), **N719** (0.0252), and **PA** (0.0299). When fabricated as devices, the **CA**, **FA**, and **PA** cells displayed similar CCTs and color differences ranging from 3729 to 3970 K and 0.0432 to 0.0469, respectively. Interestingly, the CCTs and color differences of the HCA cells were comparable to those of the **TiO**_**2**_**-only** cell, which had the values 4063 K and 0.0400, respectively. The **N719** cell, on the other hand, exhibited a lower CCT (3148 K) and a larger color shift (0.0663) from the original light source, transmitting an orangish light.

**Figure 7:**
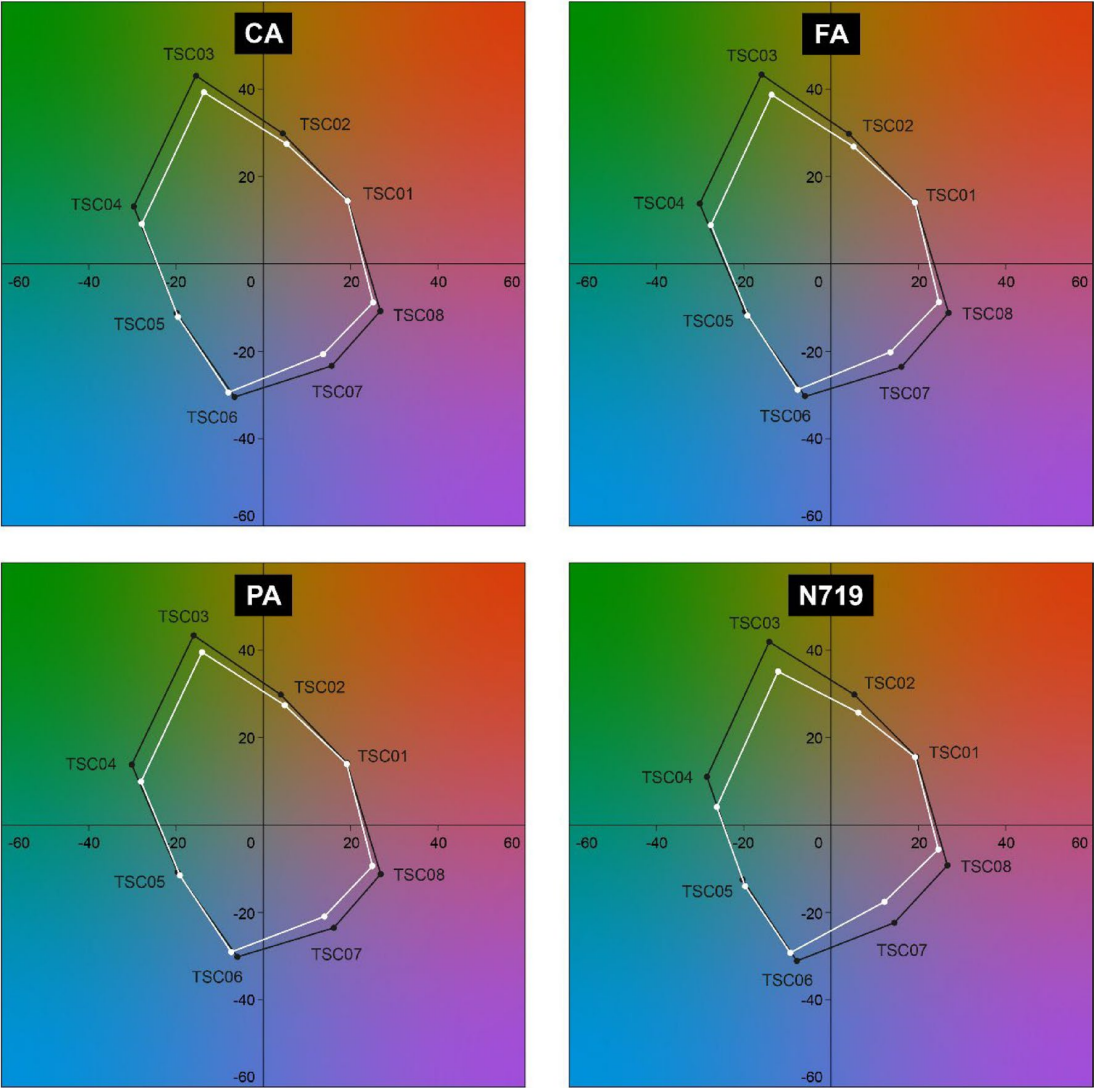
Color coordinates of the eight test color samples (TSC01 to TSC08) illuminated by light transmitted through the HCA cells (CA, FA, and PA) and the N719 cell (white dots) and the reference blackbody (black dots).

Although the light penetrated through the HCA-sensitized DSSCs is colorless, comparable to the TiO_2_-only cell, the color rendering index (CRI) should also be reported to better comprehend their color quality. Unlike the figures of merit reported above, the CRI does not consider the color of the transmitted light itself. Rather, it describes the color appearance of objects illuminated by transmitted light. The CRI of a light is the comprehensive measurement of its ability to reproduce the colors of objects using 15 test color samples (TSC01 to TSC15)^39^. It is derived by comparing the color rendering of the transmitted light to that of a reference blackbody with the same color temperature. The general CRI (*R*_a_, average of *R*_1_ to *R*_8_) was calculated by averaging the scores of the first eight color samples. These eight samples (TSC01 to TSC08) correspond to unsaturated and pastel colors. *R*_9_–*R*_15_ are special CRIs of red, yellow, green, blue, skin tone, olive green, and Asian skin colors, respectively. Table 5 lists the general CRI (*R*_a_) and the special CRIs (*R*_9_–*R*_15_) of the light penetrated through the films and devices. Figure 7 compares the color coordinates of the first eight color samples (TSC01 to TSC08) illuminated by the transmitted light of the devices and the reference blackbody. The color shift directions were plotted on the CIELAB color chart.

**Table 5:** The general CRI (*R*_a_) and the special CRIs (*R*_9_–*R*_15_) of the light transmitted through the TiO_2_-only, CA, FA, PA, and N719 photoanodic films and their dye-sensitized solar cells.

Dye	*R*_a_	*R*_9_	*R*_10_	*R*_11_	*R*_12_	*R*_13_	*R*_14_	*R*_15_
**Photoanodic films**								
TiO_2_	98	94	98	96	93	98	99	97
CA	97	90	97	95	92	97	99	95
FA	98	93	98	96	93	98	99	96
PA	95	83	94	93	88	95	99	92
N719	98	97	95	99	94	98	99	97
**Dye-sensitized solar cells**								
TiO_2_	92	76	90	88	84	93	98	88
CA	93	80	93	89	87	94	97	90
FA	90	73	88	85	79	91	97	86
PA	92	75	89	88	82	92	98	87
N719	91	75	91	84	83	93	96	88

The higher the CRI, the better the light source is at rendering colors accurately. A CRI of 100 represents the best performance of a light, while a low CRI value may result in some object colors looking unnatural and dull. Light sources with a CRI greater than 85 are considered good for color-rendering^40^. A high CRI value is especially critical for color-sensitive applications and environments where visual appearance and color accuracy are important for businesses. In general, HCA-sensitized DSSCs (**CA**, **FA**, and **PA**) and the **N719** cell exhibited excellent color-rendering ability. In terms of *R*_a_, the **FA** and **N719** cells showed slightly lower rendering abilities for TSC03, TSC04, TSC07, and TSC08 color samples compared to the **CA** and **PA** cells (see Fig. 7). The color coordinates illuminated by the **TiO**_**2**_**-only** cell are also provided in Fig. S3, confirming that there is no noticeable difference in *R*_a_ performance between the HCA cells (**CA**, **FA**, and **PA**) and the **TiO**_**2**_**-only** cell. Among the special CRIs, *R*_9_ and *R*_12_ showed low values for the HCA cells (**CA**, **FA**, and **PA**) and the **N719** cell. *R*_9_ and *R*_12_ correspond to the ability of a light source to display saturated red and blue objects, respectively. Thus, using HCA-sensitized DSSCs and **N719** cells in art galleries or museums, where red and blue hues are prevalent, requires some caution. In particular, the light transmitted from the **FA**, **PA**, and **N719** cells rendered red poorly, with notable dulling (*R*_9_ ≤ 75). Therefore, the use of these cells is not recommended in medical areas, where it is critical to accurately render the colors of tissues and blood.

## Conclusion

We investigated the spectroscopic, electrochemical, and colorimetric properties of hydroxycinnamic acid derivatives, which selectively absorb UVA light, and verified their usage as photosensitizers for UV-selective and colorless DSSCs. The DSSCs exhibited a PCE of 0.22–0.38% under AM 1.5G illumination (100 mW/cm^2^) and 3.40–3.62% under UVA (365 nm, 115.22 mW/cm^2^), with a corresponding VLT of 49.07–43.72% and *R*_*a*_ of 93–90. Commercially available derivatives of hydroxycinnamic acid are promising candidates for application in the development of wavelength-selective and visibly transparent solar cells. We are currently fabricating low-cost and large-area DSSCs with HCAs for photovoltaic windows and exploring the co-sensitization of UV- and NIR-absorbing dyes with complementary absorption spectra for panchromatic DSSCs.

## Materials and methods

### Computation details

Theoretical calculations of three hydroxycinnamic acids (caffeic acid, ferulic acid, and *p*-coumaric acid) were performed using the Gaussian 09 software package^41^. For DFT calculations, the ground-state optimized structures of the individual molecules and molecule-linked TiO_2_ were obtained at the B3LYP level^42^ with the 6-31G(d,p) basis set for C, H, O, and N atoms and the LANL2DZ basis set for the Ti atom^43^. For TDDFT calculations, the vertical electronic excitation energies and oscillator strengths for the lowest 20 transitions in the ground-state optimized structures were calculated using the CAM-B3LYP functional and 6–311 +  + G(d,p) basis set^44^. The solvation effect (ethanol, ε = 24.852) was considered for both the ground and excited states using the self-consistent reaction field (SCRF) method based on Tomasi’s polarizable continuum model (PCM)^45^.

### Materials

Caffeic acid (**CA**), ferulic acid (**FA**), *p*-coumaric acid (**PA**), and other reagents were purchased from Merck and used without further purification.

### Characterization

Photophysical and electrochemical properties of the UV absorbing compounds were determined according to our previous protocols^6^. UV–visible absorbance and transmittance spectra were recorded using a UV-2600 240 V EN spectrophotometer (Shimazu, Japan). CV was carried out at a scan rate of 100 mV/s using a potentiostat/galvanostat B22104 (CompactStat.e, Ivium Tech., Netherlands). A three-electrode cell consisting of Pt disc working electrode, Pt wire counter electrode, and Ag/Ag^+^ reference electrode was used. A ferrocene/ferrocenium (Fc/Fc^+^) couple was used as an external reference. The solutions were prepared using dry EtOH containing 0.1 M tetrabutylammonium perchlorate (TBAP) as the supporting electrolyte. The solutions were purged with nitrogen (N_2_) gas for 15 min before recording the electrochemical data.

### DSSC fabrication and photovoltaic characterization

Our DSSC fabrication procedures were motivated by the previous reports^12–15^. Transparent and conductive fluorine-doped tin oxide (FTO) glass substrates (TEC 7, Pilkington, 2.2 mm-thick, sheet resistance = 8 Ω/sq) were sequentially cleaned with acetone, 2-propanol, and deionized water, and then baked on a hot plate at 150 °C for 10 min. The conductive substrates were pre-treated with an aqueous TiCl_4_ solution (40 mM) at 75 °C for 30 min, rinsed several times with deionized water, and dried under a flow of N_2_ gas. The TiO_2_ paste (Solaronix, Ti-Nanoxide T/SP) was screen-printed onto the glass substrate and the final area of the TiO_2_ photoanode was 0.25 cm^2^. The screen-printed films were sintered at 300 °C for 30 min and then continuously at 575 °C for 1 h in a muffle furnace (KSL-1100X, MTI Corporation, USA). After cooling to room temperature (~ 20 °C) and treating O_2_ plasma, the TiO_2_ photoanodes were immersed in the aqueous TiCl_4_ solution (40 mM) at 75 °C for 30 min, rinsed with deionized water, and then heated at 550 °C for 30 min on a hot plate. The photoanodes were then dipped in a 0.5 M dye-containing EtOH solution of **CA**, **FA**, and **PA**, respectively, for 24 h. The dye concentration and dipping time were optimized for photovoltaic performance (Fig. S4). Two holes were drilled on other FTO glasses for preparing the counter electrodes (CEs). They were also ultrasonically cleaned with acetone, 2-propanol, and deionized water, and then baked at 150 °C for 10 min. A chloroplatinic acid (H_2_PtCl_6_) solution in 2-propanol was spin-coated onto the conductive substrates, followed by thermal reduction at 425 °C for 1 h in the muffle furnace. For DSSCs, the dye-grafted photoanode and Pt CE were assembled with a 25 µm-thick thermoplastic film (Surlyn™ , Solaronix, Switzerland) and then sealed by heating. An idodide-based liquid electrolyte (AN-50, Solaronix, Switzerland) was injected through the pre-drilled holes, which were then sealed with 60 µm-thick thermoplastic film (Surlyn™, Solaronix, Switzerland) and a cover glass. Five DSSCs of each dye were fabricated for photovoltaic measurements and stability tests.

The photovoltaic performance of the DSSCs were assessed according to our previous protocols^6^. The photovoltaic parameters were evaluated using a solar cell I–V measurement system (K3000 LAB, McScience, Korea) under standard illumination condition (AM 1.5G, 100 mW/cm^2^), and 365 nm UV LEDs (MK3005P, TNE Tech Co., Ltd, Korea). The external quantum efficiency (EQE) was recorded to evaluate the spectral response of the DSSCs using a solar cell EQE test system (K3100, McScience, Korea). Electrochemical impedance spectroscopy (EIS) measurements were carried out using a frequency response analyzer (CompactStat.h, Ivium Tech, Netherlands). A sinusoidal modulated AC potential with an amplitude of 10 mV was applied over a frequency range of 0.1 Hz to 500 kHz, at zero bias potential. The spectra recorded in the dark and under one-sun illumination were analyzed using an appropriate equivalent circuit model built in a complex non-linear least-square regression software (ZView®, Scribner Associates Inc., USA). The stability test was conducted by repeated photovoltaic and EIS measurements after aging.

## Supplementary Information

Supplementary Information.

## Data Availability

All data generated or analysed during this study are included in this published article and its supplementary information file.

## References

[1] United Nations Environment Programme, 2021 Global status report for buildings and construction: Towards a zero-emissions, efficient and resilient buildings and construction sector. (Nairobi, 2021).

[2] Deb SK (2001). Stand-alone photovoltaic-powered electrochromic smart window. Electrochim. Acta..

[3] Davy NC (2017). Pairing of near-ultraviolet solar cells with electrochromic windows for smart management of the solar spectrum. Nat. Energy.

[4] Liu D, Yang C, Lunt RR (2018). Halide perovskites for selective ultraviolet-harvesting transparent photovoltaics. Joule.

[5] Liu D (2019). Lead halide ultraviolet-harvesting transparent photovoltaics with an efficiency exceeding 1%. ACS Appl. Energy Mater..

[6] Marsay MA (2022). UV-harvesting dyes featuring a fluorene donor for visibly transparent and colorless dye-sensitized solar cells. Dyes Pigm..

[7] Veron AC (2014). NIR-absorbing heptamethine dyes with tailor-made counterions for application in light to energy conversion. Org. Lett..

[8] Zhang K (2014). High-performance, transparent, dye-sensitized solar cells for see-through photovoltaic windows. Adv. Energy Mater..

[9] Traverse CJ (2017). Anions for near-infrared selective organic salt photovoltaics. Sci. Rep..

[10] Naim W (2021). Transparent and colorless dye-sensitized solar cells exceeding 75% average visible transmittance. JACS Au.

[11] Grifoni F (2021). Toward sustainable, colorless, and transparent photovoltaics: state of the art and perspectives for the development of selective near-infrared dye-sensitized solar cells. Adv. Energy Mater..

[12] Yun S, Hagfeldt A, Ma T (2014). Pt-free counter electrode for dye-sensitized solar cells with high efficiency. Adv. Mater..

[13] Yun S (2018). New-generation integrated devices based on dye-sensitized and perovskite solar cells. Energy Environ. Sci..

[14] Yun S (2019). Dye sensitized photoelectrolysis cells. Chem. Soc. Rev..

[15] Lim D (2020). Blue-colored dyes featuring a diketopyrrolopyrrole spacer for translucent dye-sensitized solar cells. Dyes Pigm..

[16] Ghifari A (2020). Transparent platinum counter electrode prepared by polyol reduction for bifacial, dye-sensitized solar cells. Nanomaterials.

[17] Ludin NA (2014). Review on the development of natural dye photosensitizer for dye-sensitized solar cells. Renew. Sustain. Energy Rev..

[18] Kumara NTRN (2017). Recent progress and utilization of natural pigments in dye sensitized solar cells: a review. Renew. Sustain. Energy Rev..

[19] Iqbal MZ, Ali SR, Khan S (2019). Progress in dye sensitized solar cell by incorporating natural photosensitizers. Sol. Energy.

[20] Bengoechea L (1995). Structure of hydroxycinnamic acid derivatives established by high-performance liquid chromatography with photodiode-array detection. Chromatographia.

[21] Robards K (1999). Phenolic compounds and their role in oxidative processes in fruits. Food Chem..

[22] Escarpa A, Gonzalez MC (2001). An overview of analytical chemistry of phenolic compounds in foods. Crit. Rev. Anal. Chem..

[23] Kowalski R, Kowalska G (2005). Phenolic acid contents in fruits of aubergine (*Solanum Melongena* L.). Pol. J. Food Nutr. Sci..

[24] Mazzone G, Russo N, Toscano M (2016). Antioxidant properties comparative study of natural hydroxycinnamic acids and structurally modified derivatives: computational insights. Comput. Theor. Chem..

[25] Beneduci A, Furia E, Russo N, Marino T (2017). Complexation behaviour of caffeic, ferulic and p-coumaric acid towards aluminum cations: a combined experimental and theoretical approach. New J. Chem..

[26] Catauro M, Barrino F, Poggetto GD, Crescente G, Piccolella S, Pcifico S (2020). New SiO_2_/caffeic acid hybrid materials: synthesis, spectroscopic characterization, and bioactivity. Materials.

[27] Bartolome B (1993). Photodiode array detection for elucidation of the structure of phenolic compounds. J. Chromatogr. A.

[28] Persson P, Bergstrom R, Lunell S (2000). Quantum chemical study of photoinjection processes in dye-sensitized TiO_2_ nanoparticles. J. Phys. Chem. B.

[29] Zhang L, Cole JM (2015). Anchoring groups for dye-sensitized solar cells. ACS Appl. Mater. Interfaces.

[30] Sowmiya M, Senthilkumar K (2017). Opto-electronic and interfacial charge transfer properties of azobenzene dyes on anatase TiO_2_ (001) surface–the effect of anchoring group. J. Photochem. Photobiol. A.

[31] Kontos AG (2013). Long-term thermal stability of liquid dye solar cells. J. Phys. Chem. C.

[32] ISO, Glass in building-Determination of light transmittance, solar direct transmittance, total solar energy transmittance, ultraviolet transmittance and related glazing factors, ISO 9050:2003 (2003).

[33] Kang JS (2018). Highly efficient bifacial dye-sensitized solar cells employing polymeric counter electrodes. ACS Appl. Mater. Interfaces.

[34] Carter, E. C. *et al*. CIE 15-2018 Colorimetry. (International Commission on Illumination, Vienna, 2018).

[35] Boynton, R. M. *et al*. CIE 75-1988 Spectral luminous efficiency functions based upon brightness matching for monochromatic point sources with 2° and 10° fields. (International Commission on Illumination, Vienna, 1988).

[36] Schanda J (2007). Colorimetry: Understanding the CIE System.

[37] Boyce PR (2003). Human Factors in Lighting.

[38] Lindsey JL (1997). Applied Illumination Engineering.

[39] Azuma, T. *et al*. CIE 13.3-1995 Method of measuring and specifying colour rendering properties of light sources. (International Commission on Illuminations, Vienna, 1995).

[40] Christopher CJ, Pandey R, Barr MC, Lunt RR (2017). Emergence of highly transparent photovoltaics for distributed applications. Nat. Energy.

[41] Frisch M (2009). Gaussian 09, Revision d. 01.

[42] Lee C, Yang W, Parr RG (1988). Development of the Colle-Salvetti correlation-energy formula into a functional of the electron density. Phys. Rev..

[43] Oprea CI (2013). Density functional theory (DFT) study of coumarin-based dyes adsorbed on TiO_2_ nanoclusters-applications to dye-sensitized solar cells. Materials.

[44] Rodrigues-Oliveira AF, Ribeiro FWM, Cervi G, Correra TC (2018). Evaluation of common theoretical methods for predicting infrared multiphotonic dissociation vibrational spectra of intramolecular hydrogen-bonded ions. ACS Omega.

[45] Tomasi J, Cammi R (2005). Quantum mechanical continuum solvation models. Chem. Rev.

