# Supplementary material for: Hydroxycinnamic acid derivatives for UV-selective and visibly transparent dye-sensitized solar cells
Source: Sci Rep. 2023 Feb 24;13:3235. doi: 10.1038/s41598-022-17236-6 (PMC9958184; doi:10.1038/s41598-022-17236-6)
Supplement: Supplementary file 1 — Supplementary Information. [file 41598_2022_17236_MOESM1_ESM.docx]

(Electronic Supporting Information)

**Hydroxycinnamic Acid Derivatives for UV-selective and Visibly Transparent Dye-Sensitized Solar Cells**

Arum Dista Wulansari^1^, Dini Hayati^1^, Dang Xuan Long^2^, Kyungah Choi^3,*^, Jongin Hong^1,2,*^

^1^ Department of Chemistry, Chung-Ang University, 84 Heukseok-ro, Dongjak-gu, Seoul 06975, Republic of Korea

^2^ Department of Smart Cities, Chung-Ang University, 84 Heukseok-ro, Dongjak-gu, Seoul 06974, Republic of Korea

^3^ Department of Interior Architecture Design, Hanyang University, 222 Wangsimni-ro, Seongdong-gu, Seoul 04763, Republic of Korea

* Corresponding authors: kchoi@hanyang.ac.kr (K. Choi) and hongj@cau.ac.kr (J. Hong)

**Table S1.** Calculated adsorption energies (*E_ads_*) between a dye and (TiO_2_)_9_

| CA(catechol)-(TiO_2_)_9_ | CA-(TiO_2_)_9_ | FA-(TiO_2_)_9_ | PA-(TiO_2_)_9_ |
| --- | --- | --- | --- |
| -1.568 eV | -0.775 eV | -0.748 eV | -0,738 eV |

$E_{ads}= E_{Dye-{TiO}_{2}}-E_{{TiO}_{2}}-E_{Dye}$ where $E_{Dye-{TiO}_{2}}$, $E_{{TiO}_{2}}$, $E_{Dye}$, are the total energy of the dye-grafted TiO_2_, total energy of the TiO_2_ surface and total energy of the isolated dye, respectively.

**Table S2.** Photovoltaic performance of DSSCs sensitized with CA, FA, and PA under UVA irradiation (365 nm) as a function of UVA intensity.

| **Sample** | **P(mW/cm^2^)** | ***V*_oc_ (V)** | ***J*_sc_ (mA/cm^2^)** | **FF(%)** | ***η*(%)** |
| --- | --- | --- | --- | --- | --- |
| TiO_2_ only | 57.6 | 0.656 | 3.01 | 60.74 | 2.08 |
|  | 115.2 | 0.696 | 8.18 | 54.85 | 2.71 |
|  | 172.8 | 0.696 | 14.14 | 52.82 | 3.01 |
|  | 230.4 | 0.686 | 20.66 | 51.87 | 3.19 |
|  | 288.1 | 0.673 | 26.68 | 51.55 | 3.21 |
| CA | 57.6 | 0.621 | 5.38 | 59.40 | 3.44 |
|  | 115.2 | 0.642 | 11.68 | 55.62 | 3.62 |
|  | 172.8 | 0.648 | 18.13 | 52.10 | 3.54 |
|  | 230.4 | 0.646 | 24.60 | 49.17 | 3.39 |
|  | 288.1 | 0.636 | 30.71 | 46.84 | 3.17 |
| FA | 57.6 | 0.608 | 4.97 | 60.96 | 3.20 |
|  | 115.2 | 0.662 | 10.54 | 56.97 | 3.45 |
|  | 172.8 | 0.638 | 16.75 | 54.03 | 3.34 |
|  | 230.4 | 0.634 | 22.73 | 51.21 | 3.20 |
|  | 288.1 | 0.623 | 28.38 | 48.97 | 3.00 |
| PA | 57.6 | 0.618 | 4.75 | 62.21 | 3.17 |
|  | 115.2 | 0.631 | 10.79 | 57.48 | 3.40 |
|  | 172.8 | 0.676 | 16.62 | 53.52 | 3.48 |
|  | 230.4 | 0.678 | 22.87 | 50.69 | 3.41 |
|  | 288.1 | 0.673 | 29.07 | 48.09 | 3.27 |

**Table S3.** Photovoltaic performance of DSSCs sensitized with CA, FA, and PA under one-sun illumination after a period of aging at room temperature.

| Sample | Aging(Day) | *V*_oc_ (V) | *J*_sc_ (mA/cm^2^) | FF(%) | PCE(%) |
| --- | --- | --- | --- | --- | --- |
| TiO_2_ only | 0 | 0.463±0.003 | 0.05±0.26 | 86.58±0.63 | 0.020±0.001 |
|  | 2 | 0.442±0.001 | 0.04±0.01 | 84.71±0.01 | 0.016±0.085 |
|  | 4 | 0.428±0.002 | 0.04±0.01 | 80.22±0.01 | 0.015±0.001 |
|  | 6 | 0.365±0.001 | 0.04±0.01 | 78.31±0.01 | 0.012±0.063 |
|  | 8 | 0.309±0.004 | 0.04±0.01 | 76.66±0.01 | 0.010±0.001 |
|  | 10 | 0.301±0.004 | 0.04±0.01 | 76.06±0.02 | 0.009±0.001 |
| CA | 0 | 0.545±0.003 | 1.00±0.02 | 69.24±0.69 | 0.38±0.01 |
|  | 2 | 0.505±0.003 | 0.97±0.01 | 67.22±0.22 | 0.37±0.01 |
|  | 4 | 0.474±0.012 | 0.93±0.01 | 64.38±0.22 | 0.35±0.01 |
|  | 6 | 0.469±0.002 | 0.85±0.01 | 60.95±0.021 | 0.32±0.01 |
|  | 8 | 0.393±0.018 | 0.62±0.02 | 58.17±0.38 | 0.31±0.01 |
|  | 10 | 0.391±0.005 | 0.50±0.01 | 57.51±0.49 | 0.29±0.01 |
| FA | 0 | 0.536±0.002 | 0.93±0.01 | 67.68±0.36 | 0.34±0.01 |
|  | 2 | 0.522±0.001 | 0.84±0.01 | 62.56±0.01 | 0.32±0.01 |
|  | 4 | 0.496±0.001 | 0.82±0.01 | 62.16±0.05 | 0.31±0.01 |
|  | 6 | 0.472±0.001 | 0.74±0.01 | 53.84±0.01 | 0.30±0.02 |
|  | 8 | 0.461±0.001 | 0.32±0.01 | 53.23±0.58 | 0.27±0.01 |
|  | 10 | 0.393±0.004 | 0.22±0.01 | 46.84±0.01 | 0.24±0.01 |
| PA | 0 | 0.532±0.005 | 0.63±0.01 | 67.46±0.49 | 0.22±0.01 |
|  | 2 | 0.501±0.013 | 0.62±0.01 | 63.15±0.01 | 0.21±0.01 |
|  | 4 | 0.491±0.002 | 0.60±0.01 | 62.75±0.01 | 0.20±0.01 |
|  | 6 | 0.489±0.004 | 0.57±0.01 | 60.29±0.01 | 0.15±0.04 |
|  | 8 | 0.482±0.001 | 0.56±0.02 | 56.22±0.01 | 0.12±0.01 |
|  | 10 | 0.433±0.001 | 0.42±0.01 | 54.61±0.01 | 0.09±0.01 |

**Table S4.** Photovoltaic performance of DSSCs sensitized with CA, FA, and PA under UVA irradiation (365 nm) after a period of aging at room temperature.

| Sample | Aging(Day) | *V*_oc_ (V) | *J*_sc_ (mA/cm^2^) | FF(%) | PCE(%) |
| --- | --- | --- | --- | --- | --- |
| TiO_2_ only | 0 | 0.696±0.004 | 8.18±0.03 | 54.85±0.69 | 2.71±0.04 |
|  | 2 | 0.671±0.001 | 8.06±0.01 | 54.84±0.01 | 2.45±0.01 |
|  | 4 | 0.665±0.001 | 7.85±0.01 | 54.71±0.01 | 2.17±0.01 |
|  | 6 | 0.522±0.003 | 6.81±0.01 | 50.69±0nm,./.01 | 1.75±0.01 |
|  | 8 | 0.506±0.001 | 3.45±0.01 | 42.69±0.01 | 1.71±0.01 |
|  | 10 | 0.489±0.001 | 2.28±0.01 | 40.20±0.10 | 1.62±0.01 |
| CA | 0 | 0.642±0.073 | 11.68±0.95 | 55.62±0.61 | 3.62±0.03 |
|  | 2 | 0.635±0.001 | 10.64±0.01 | 53.79±0.02 | 3.43±0.01 |
|  | 4 | 0.632±0.002 | 10.60±0.31 | 53.06±0.02 | 3.33±0.01 |
|  | 6 | 0.621±0.001 | 10.20±0.09 | 52.85±0.01 | 3.24±0.02 |
|  | 8 | 0.607±0.001 | 7.91±0.02 | 50.91±0.01 | 3.17±0.01 |
|  | 10 | 0.605±0.001 | 7.17±0.02 | 49.80±0.01 | 3.05±0.01 |
| FA | 0 | 0.662±0.011 | 10.54±0.15 | 56.97±0.53 | 3.45±0.02 |
|  | 2 | 0.661±0.001 | 10.45±0.01 | 56.77±0.01 | 3.31±0.01 |
|  | 4 | 0.643±0.001 | 10.37±0.01 | 55.01±0.01 | 3.20±0.02 |
|  | 6 | 0.601±0.001 | 9.16±0.01 | 54.80±0.08 | 3.15±0.01 |
|  | 8 | 0.545±0.001 | 8.30±0.07 | 53.77±0.01 | 3.02±0.01 |
|  | 10 | 0.503±0.050 | 4.68±0.01 | 51.37±0.01 | 2.95±0.01 |
| PA | 0 | 0.631±0.087 | 10.79±0.12 | 57.48±0.40 | 3.40±0.08 |
|  | 2 | 0.629±0.002 | 9.39±0.03 | 57.45±0.18 | 3.30±0.01 |
|  | 4 | 0.623±0.003 | 8.82±0.01 | 57.07±0.01 | 3.12±0.02 |
|  | 6 | 0.616±0.001 | 8.32±0.03 | 50.27±0.01 | 3.10±0.01 |
|  | 8 | 0.614±0.001 | 7.91±0.01 | 49.81±0.01 | 3.05±0.01 |
|  | 10 | 0.559±0.001 | 7.21±0.06 | 49.23±0.02 | 2.87±0.03 |

**Figure S1.**

**
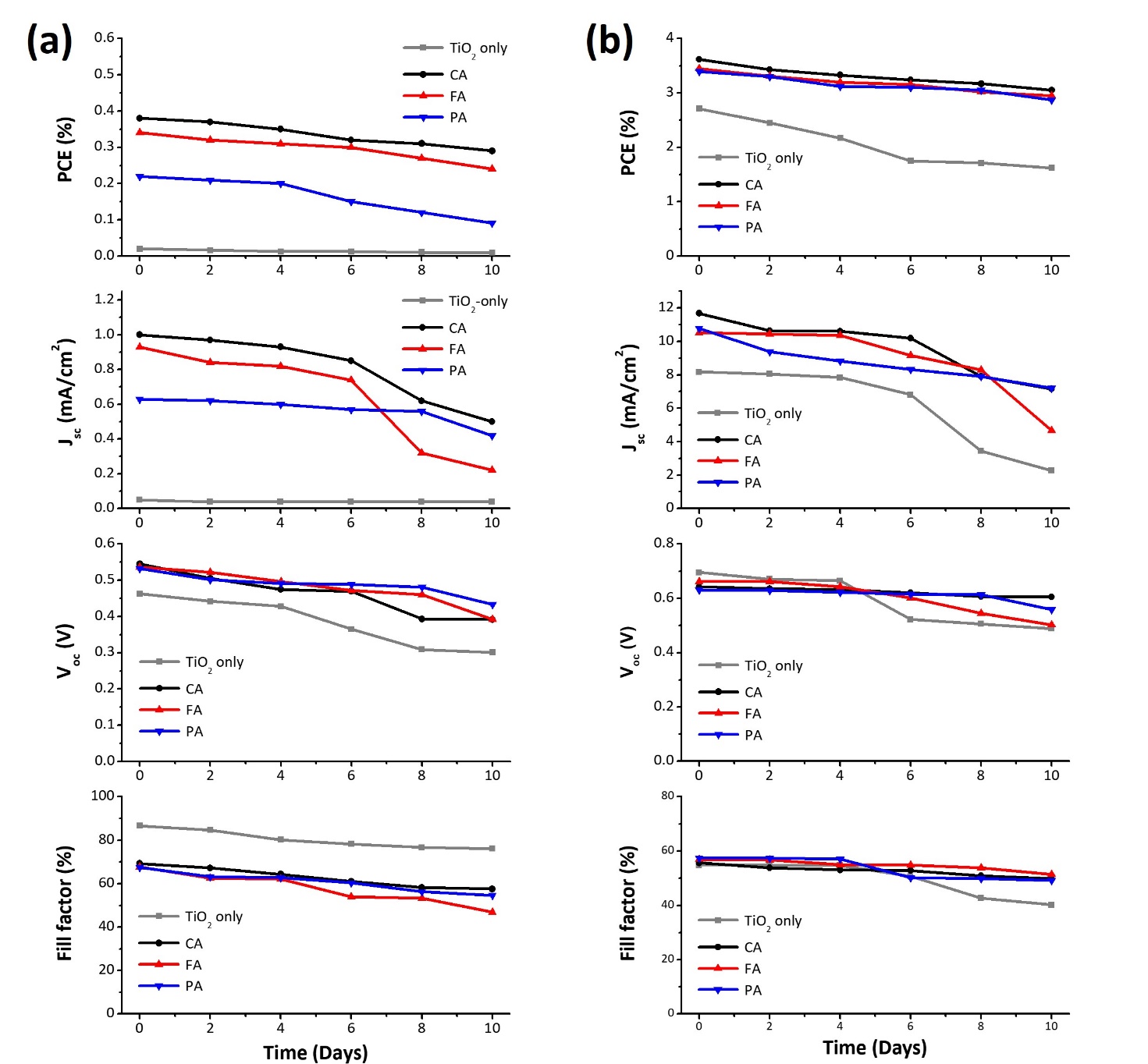
**

**Figure S1.** Efficiencies (PCE), short circuit currents (*J_sc_*), open circuit voltages (*V_oc_*), and fill factors of the DSSCs, periodically assessed at (a) 1 sun and (b) UVA, after a period of aging at room temperature and in an open circuit.

**Figure S2.**

**
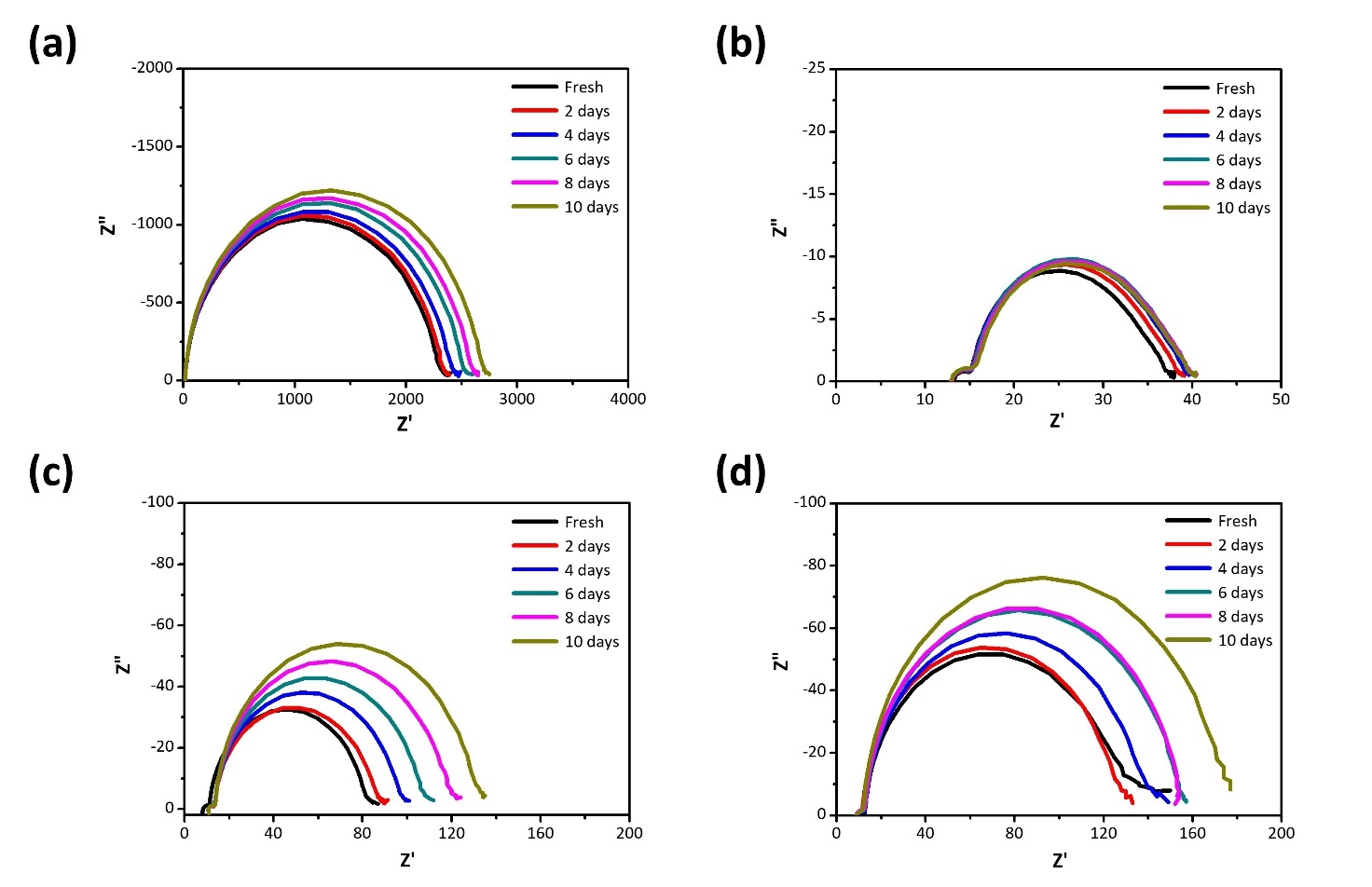
**

**Figure S2.** EIS stability tests at 0V from 0.1 Hz to 500 kHz on the DSSCs: (a) TiO_2_ only, (b) CA, (c) FA, and (d) PA in a dark.

**Figure S3.**


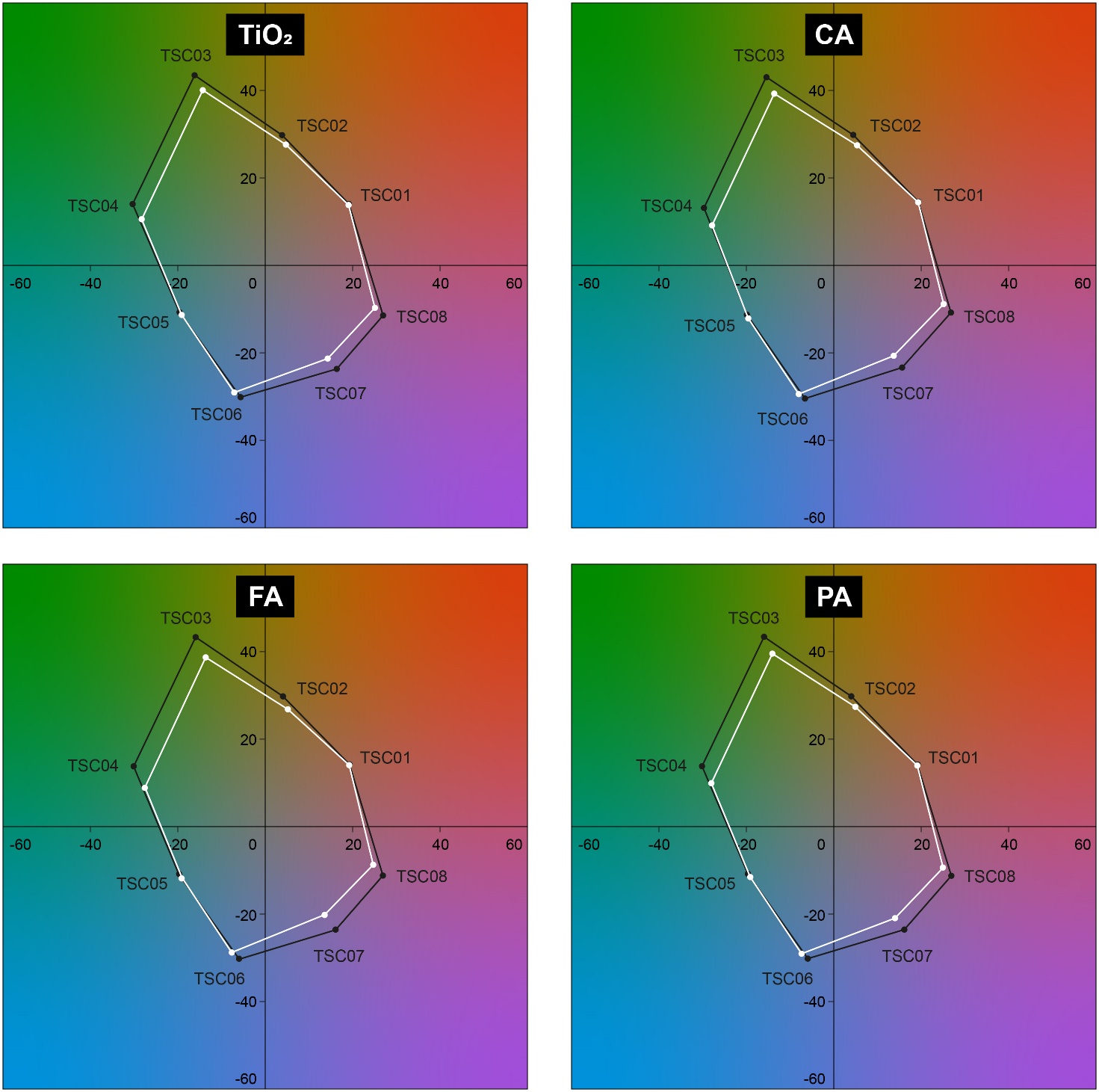


**Figure S3.** Color coordinates of the eight test color samples (TSC01 to TSC08) illuminated by the light transmitted through the devices (white dots) and the reference black body (black dots). Plotted are the data for the HCA cells (**CA**, **FA**, and **PA**) and the N719 cell.

**Figure S4.**


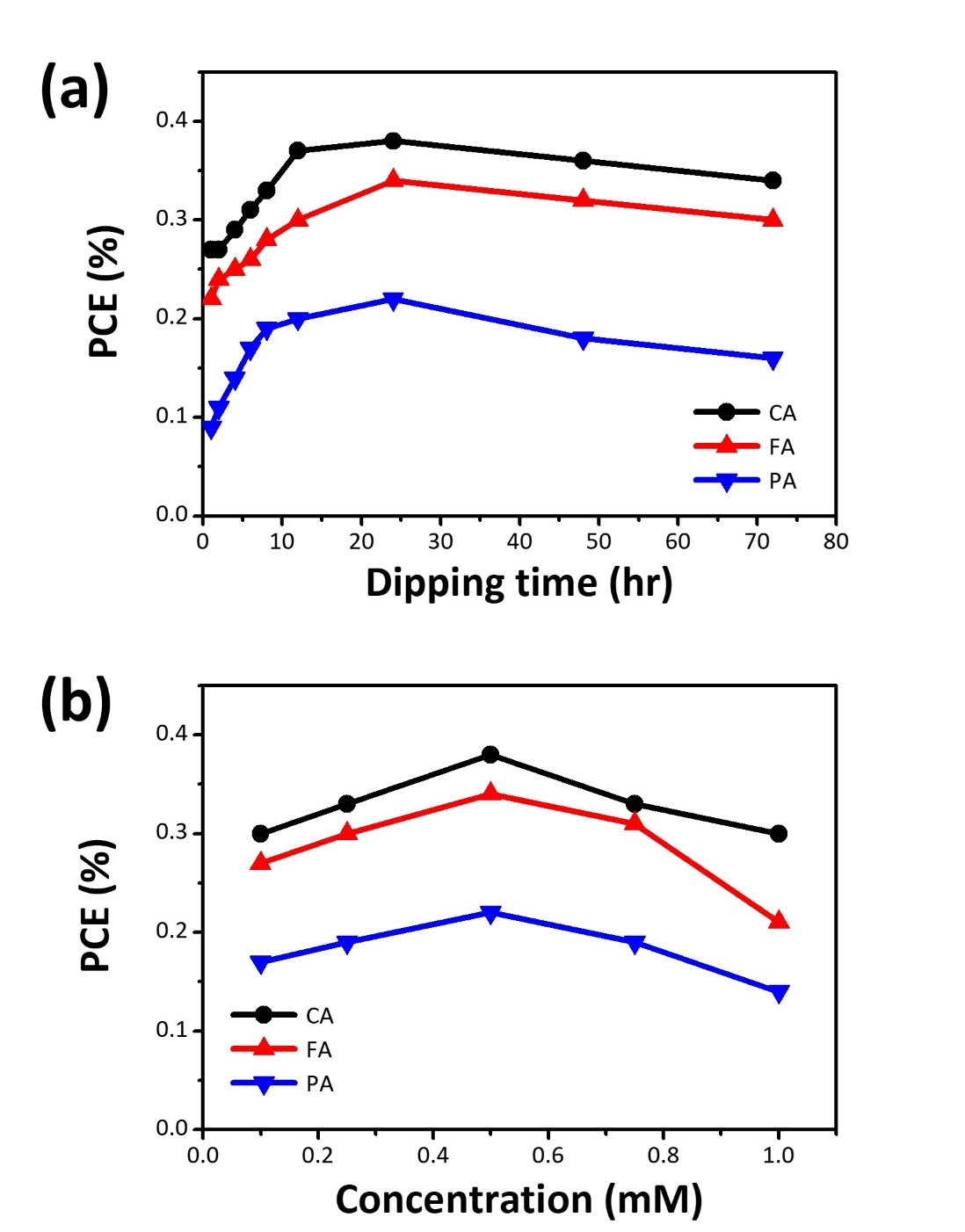


**Figure S4.** Power conversion efficiencies of the HCA cells as a function of (a) dye dipping time and (b) dye solution concentration.
